# Structure of boehmite-derived γ-alumina and its transformation mechanism revealed by electron crystallography

**DOI:** 10.1107/S2052520621008027

**Published:** 2021-09-16

**Authors:** Zhiping Luo

**Affiliations:** a Department of Chemistry, Physics and Materials Science, Fayetteville State University, Fayetteville, North Carolina 28301, USA

**Keywords:** alumina, boehmite, crystal structure, electron diffraction, quantitative microscopy, transformation mechanism

## Abstract

γ-Alumina is a widely used material, while its precise crystal structure and transformation mechanism derived from boehmite have remained unclear in the literature for decades. In this work, quantitative electron microscopy has been applied to study the crystalline structure of γ-alumina and its transformation mechanism from boehmite. Based on Rietveld refinement of electron diffraction patterns, a new tetragonal structure model, with a space group of *I*4_1_/*amd* (No. 141), was proposed for the γ-alumina phase, with Al cations on 4*a*, 8*c*, 8*d* and 16*g* sites and O anions on the 16*h* site, which could provide better fits than current models. During the boehmite to γ-alumina transformation induced by e-beam irradiation, when the boehmite layers were oriented along the edge-on direction, a shrinkage caused by dehydration was directly observed. Two kinds of boehmite to γ-alumina transformation mechanisms, namely collapse and reaction mechanisms, were elucidated crystallographically in detail with new insights through an intermediate structure, and the reaction mechanism was demonstrated to produce much reduced changes in dimensions and volume, compared with the collapse mechanism. The experimental observations supported the reaction mechanism, which occurred through partial occupation of the dehydrated space by diffusion in the initial stage of the transformation, without the formation of voids that only appeared after the initial stage. Filling tetrahedral interstices of the intermediate structure with Al cations in different ways yields tetragonal or cubic γ-alumina structures, and the tetragonal structure is energetically favorable because of smaller lattice distortions required, compared with the cubic structure. The crystallographic orientation relationships of γ-alumina with the parent boehmite phase deduced from the proposed mechanisms are consistent with the experimental observations.

## Abstract 2

The crystal structure of γ-alumina was revealed by electron crystallography and its transformation mechanism from boehmite is proposed.

## 1. Introduction   

Alumina, or aluminium oxide (Al_2_O_3_), is an important ceramic material because of its distinct physical properties, such as low density, high porosity and high specific surface area, high modulus, high melting point, and low thermal conductivity (Poco *et al.*, 2001 ▸). Among its polymorphs, α-Al_2_O_3_ (corundum) is a thermodynamically stable phase, which is formed from aluminium oxyhydroxide AlOOH (boehmite) in a thermal transformation sequence as follows (Ingram-Jones *et al.*, 1996 ▸; Boumaza, Favaro, Ledion *et al.*, 2009 ▸):
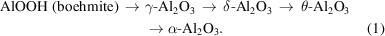
Here, γ-, δ- and θ-Al_2_O_3_ are metastable transitional alumina forms. Among these polymorphs, γ-Al_2_O_3_ is the most widely studied alumina because of its wide applications for sorbents, support for catalysts, and materials used in refining and petrochemical industries (Trueba & Trasatti, 2005 ▸; Cai & Yu, 2007 ▸; Mandić *et al.*, 2020 ▸; Batista *et al.*, 2020 ▸).

However, the precise crystalline structure of the γ-Al_2_O_3_ phase is still the subject of scientific debate (Prins, 2020 ▸; van Gog, 2021 ▸). As recently pointed out by Prins (2020 ▸), the main reason is that it has not proved possible to prepare macrocrystalline γ-Al_2_O_3_, and only nanocrystalline γ-Al_2_O_3_ has been prepared, limiting the structure determination by X-ray (XRD) or neutron diffraction (ND) techniques because of peak broadening. The following structural models have been proposed.

(*a*) Cubic structure. The γ-Al_2_O_3_ phase was conventionally described as an MgAl_2_O_4_-type spinel structure (Verwey, 1935 ▸; Lippens & de Boer, 1964 ▸; Wilson, 1979 ▸; Gutiérrez *et al.*, 2002 ▸). The MgAl_2_O_4_ spinel structure has the space group 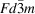, with atoms of Mg at 8*a*, Al at 16*d*, and O at 32*e* sites. Zhou & Snyder (1991 ▸) refined its structure using XRD and ND by adding a 32*e* site to the spinel structure, *i.e.* Al at 8*a* and 16*d* (spinel sites) and 32*e* (nonspinel site). The O atom is at 32*e* with occupation of 1.0, while the cationic positions are partially occupied to maintain the Al_2_O_3_ stoichiometry. In addition, Paglia *et al.* (2003 ▸) proposed and tested a cubic 16*c* structure, with the addition of 16*c* to the spinel structure. On the other hand, Smrčok *et al.* (2006 ▸) synthesized whiskers of γ-alumina single crystals in a nonstandard route using a mixture of β-sialon powder and 20 wt% of steel sawdust that were homogenized in a planetary ball mill, followed with heating to a very high temperature of 1973 K for solid-state reactions to form iron silicides and whiskers of γ-alumina. The single-crystal X-ray diffraction refinement revealed Al at 8*a*, 16*c* (nonspinel), 16*d* and 48*f* (nonspinel) sites.

(*b*) Tetragonal structure. The tetragonal distortion from a perfect cubic structure has been indeed noticed for a quite long time (Lippens & de Boer, 1964 ▸; Wilson, 1979 ▸), and the *c*/*a* ratio was found to be less than 1 in the range of 0.981–0.993 (Wilson, 1979 ▸). Paglia *et al.* (2003 ▸, 2004 ▸, 2005 ▸) conducted ND and transmission electron microscopy (TEM) of γ-alumina. They compared the pure spinel model, Zhou–Snyder model and cubic 16*c* model with a tetragonal model, and found a better fit could be obtained using the tetragonal structure model with space group *I*4_1_/*amd*, which is composed of Al at 4*a*, 8*c* and 8*d*, and O at 16*h* (Paglia *et al.*, 2003 ▸).

(*c*) Monoclinic structure. Based on density functional theory (DFT) computation, Pinto *et al.* (2004 ▸) theoretically predicted the γ-Al_2_O_3_ structure as monoclinic with space group *C*2/*m*, which is composed of purely spinel positions. Digne *et al.* (2004 ▸) described a different monoclinic structure model with a space group of *P*2_1_/*m*, obtained from previous DFT calculations by the same group (Krokidis *et al.*, 2001 ▸), which contains a significant fraction of nonspinel positions.

In addition to the discrepancies in the crystal structure determination of γ-Al_2_O_3_, its transformation mechanism from boehmite through dehydration is also not clear. Basically, the following transformation mechanisms have been proposed.

(*a*) Collapse mechanism. Since the boehmite contains Al—O—OH layers with hydrogen between these layers (Christensen *et al.*, 1982 ▸; Bokhimi *et al.*, 2001 ▸), it is intuitive to consider a collapse of layers after dehydration by removing H_2_O molecules (Lippens & de Boer, 1964 ▸; Krokidis *et al.*, 2001 ▸) or H_2_ gas under high-energy irradiation (Westbrook *et al.*, 2015 ▸; Kaddissy *et al.*, 2017 ▸, 2019 ▸; LaVerne & Huestis, 2019 ▸). In an early pioneer work, Lippens & de Boer (1964 ▸) studied the transformation of boehmite to γ-alumina and others by selected-area electron diffraction (SAED). The transformation orientation relationship (OR) was observed as
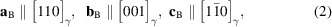
where the subscripts B and γ represent boehmite and the γ phase, respectively, and the γ phase is described as a cubic spinel structure. The predicted shrinkage is 31%, which is very large after the dehydration. This collapse mechanism was also proposed for other similar structures, such as FeOOH (Desiraju & Rao, 1982 ▸), CoOOH (Figlarz *et al.*, 1976 ▸) and CdOOH (Niepce *et al.*, 1977 ▸).

Based on DFT calculations, Krokidis *et al.* (2001 ▸) reported a theoretical study of the dehydration process from boehmite to γ-alumina. The transformation process involves multiple steps as follows: (i) dehydration by hydrogen transfer and H_2_O extraction; (ii) structural collapse accompanied with structural shearing to form a monoclinic structure; and (iii) Al migration from octahedral to tetrahedral sites to form the γ-alumina structure. This mechanism involves both collapse and diffusion processes. Nguefack *et al.* (2003 ▸) further discussed the mechanisms by considering full dehydration and partial dehydration, and obtained two different quadratic cells. Very recently, van Gog (2021 ▸) conducted a DFT study of the dehydration interface between boehmite and γ-Al_2_O_3_, and identified interface defects during relaxation. To date, no direct experimental observations were reported to support this collapse mechanism.

(*b*) Reaction mechanism. Another pioneering work on the boehmite to γ-Al_2_O_3_ transformation was made by Wilson (1979 ▸) using X-ray and SAED. *In situ* transformation by e-beam irradiation from boehmite to γ-Al_2_O_3_ was observed, with the formation of lamellar pores and {111}_γ_ lattice fringes. As no shrinkage was observed, the transformation mechanism was proposed based on a diffusion process, involving counter­migration of protons and Al^3+^ cations, with a constraint that the direction of diffusion is governed by the hydrogen bond chain in boehmite. This process results in the formation of voids or pores, without the need for layers to collapse. This mechanism was also proposed for other structures, such as Mg(OH)_2_ (Ball & Taylor, 1961 ▸). To date, no experimental results are reported to support this reaction mechanism.

So far, the research on alumina is predominately conducted experimentally using XRD or ND, or theoretically. Recently, Ayoola, House *et al.* (2020 ▸) conducted a comparison of these current structure models using single-crystal SAED. It was found that the SAED spot pattern symmetry suggested that both the cubic Smrčok spinel model and the tetragonal Paglia model are better than the monoclinic models; further, the former traditional cubic spinel model is more accurate than the latter tetragonal model.

As the alumina materials are in the nano form, the electron microscopy method may have some advantages over the traditional X-ray or neutron methods, because of the strong interaction of electrons with the material, so only minute crystals are needed to avoid any impurity phases, and *in situ* transformation by e-beam irradiation enables a direct observation through imaging. Whereas the electron multiple scattering effect could be ignored in the quantification of nanocrystals if they are well dispersed without aggregation along the electron beam direction (Weirich *et al.*, 1996 ▸; Weirich, Winterer *et al.*, 2000 ▸; Weirich, Zou *et al.*, 2000 ▸; Weirich *et al.*, 2002 ▸; Li, 2012 ▸; 2018 ▸). In this work, we attempted to conduct quantitative refinements of the electron diffraction patterns, for the first time, to reveal the crystal structure of γ-alumina in a different approach over the traditional methods. Through *in situ* observation by e-beam irradiation of the boehmite → γ-alumina transformation, a crystallographic relationship was established and dimensional changes were recorded. Based on the experimental results, we discuss the boehmite → γ-alumina transformation mechanism, providing a clear crystallographic picture to illustrate how these two types of transformation mechanisms occur, involving the formation of different γ-alumina structure models.

## 2. Experimental methods   

The experimental materials were commercial nanoparticles provided by Sasol, namely CATAPAL 200 boehmite and γ-alumina nanoparticles. The sample synthesis is described in the literature (Baxter, 2005 ▸; Sasol, 2021 ▸) and the γ-alumina was synthesized by calcinating boehmite at 500−800 °C. Nanoparticles were deposited on carbon film supported grids. TEM work was conducted at room temperature using a JEOL 2010 TEM instrument at 200 kV, which was equipped with a Gatan SC1000 ORIUS CCD camera (4008 × 2672 pixels). In order to record electron diffraction patterns accurately, the camera length in diffraction mode was kept constant, to avoid any error due to the presence of magnetic hysteresis in the microscope. At first, a commercial standard of Al foil was used to take polycrystalline electron diffraction ring patterns for correcting any system distortions and calibrating the camera constant. In the next step, the sample was loaded into the microscope, while any necessary focusing was made by moving the specimen *Z*-height mechanically, without using the electrical focus knob that interferes with the optical focal length. The SAED pattern intensity profiles were produced using the *ELD* program (Zou *et al.*, 1993*a* ▸,*b* ▸) in the *CRISP* package (Hovmöller, 1992 ▸). Some of the patterns were processed using *DiffTools* which enables calibration and finding zero point (Mitchell, 2008 ▸). However, even with careful calibration, slight errors in the scale (camera length) or zero point could still exist, which is one of the two major weaknesses of the electron diffraction method, compared with X-ray or neutron diffraction (another weakness is the high background which will be discussed in the following section). Therefore, the patterns were further calibrated using the diffraction peaks from the known boehmite phase as an internal standard through a linear relationship
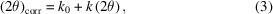
where *k*_0_ is the zero-point correction, and *k* is the scale correction. After the calibration, the *Reflex* module in *Materials Studio* by BIOVIA was used to conduct Rietveld refinement and pattern processing, as described previously (Luo *et al.*, 2011 ▸). In the Rietveld refinement algorithm, according to the *Reflex* manual, the profile residual factor *R*_p_, weighted profile residual *R*_wp_ and background-corrected weighted profile residual *R*_wpb_ are defined as
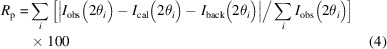


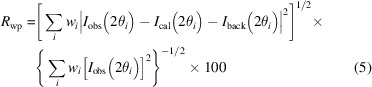


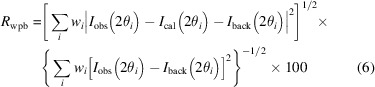
where *I*_back_ is the background intensity and *w*_*i*_ is a weighting function.

To get accurate size measurement, imaging magnifications were calibrated using SiC lattice fringes at different nominal magnifications (Luo, 2006 ▸). Simulation of single diffraction patterns were made using the *Tempas* program. Stereo projections were plotted using *PTCLab* (Gu *et al.*, 2016 ▸), and crystal structures are illustrated using the *VESTA* program (Momma & Izumi, 2011 ▸).

## 3. Results and discussion   

### 3.1. Structure of synthetic γ-alumina   

In order to analyze the electron diffraction patterns of γ-alumina, we first simulate the electron diffraction patterns of cubic Smrčok, tetragonal Paglia, monoclinic Digne and Pinto models, as shown in Fig. S1(*a*)–S1(*d*), respectively. Overall, most of their peak positions are consistent, especially the patterns from the Smrčok and Paglia models are similar; while the two monoclinic models are characterized with many peaks due to their lower symmetries, and the Digne model produces a strong peak (100) at a lower angle. As the peak intensities may vary upon the atomic occupancy and coordination deviation from the model, structural refinement is needed to fit the intensity.

**Figure 1:**
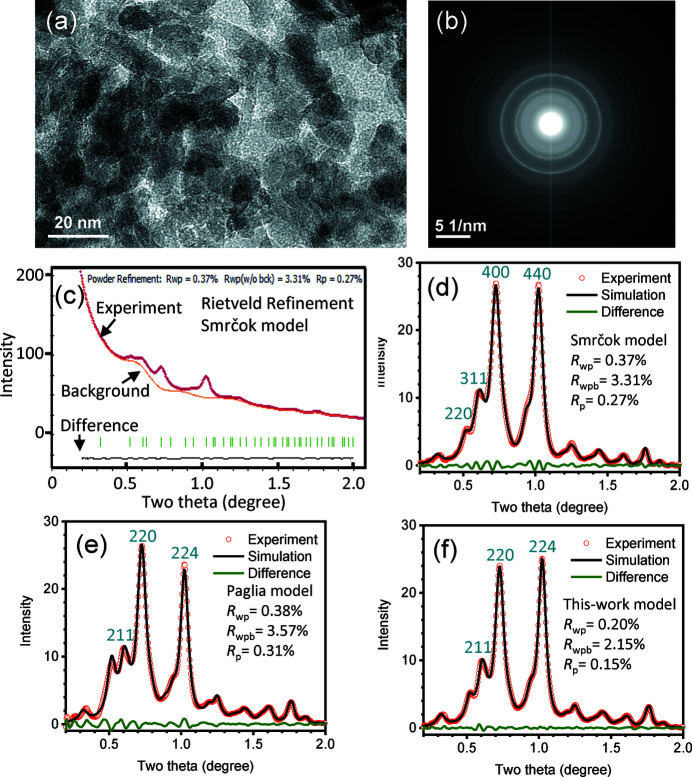
For synthetic γ-alumina nanoparticles: (*a*) TEM image, (*b*) electron diffraction image, (*c*) intensity profile of (*b*) and Rietveld refinement using Smrčok model, (*d*) Rietveld refined profile using Smrčok model after background removal and (*e*) Rietveld refined profile using Paglia model after background removal; (*f*) Rietveld refined profile using this-work model after background removal.

Fig. 1 ▸(*a*) shows an image of commercial synthetic γ-alumina nanoparticles (∼10 nm) dispersed on carbon support film. The SAED pattern is shown in Fig. 1 ▸(*b*) with polycrystalline rings. Its intensity profile is plotted in Fig. 1 ▸(*c*). Using this intensity profile, Rietveld refinements are conducted using different cubic and tetragonal models [Fig. S2 and Figs. 1 ▸(*d*)–1 ▸(*f*)] and some results are listed in Table 1 ▸. The high background starting from the center beam containing contributions from amorphous carbon could be refined (Czigany & Hultman, 2010 ▸), as indicated in Fig. 1 ▸(*c*). After subtracting the experimental intensity profile from the refined background, the observed and simulated profiles are obtained, in a similar way to XRD or ND patterns, while these profiles are dependent on the refined background using the structure model, even though the experimental diffraction pattern is the same. For example, the profiles in Figs. 1 ▸(*d*), 1 ▸(*e*) and 1 ▸(*f*) are slightly different because of their different refined background profiles

First, we compare the results of cubic systems. In the refinement of electron diffraction patterns, the weighted profile factor *R*_wp_ and profile factor *R*_p_ are typically low because of the high background contribution, since the observed intensity *I*_obs_ containing background is in the denominators of *R*_p_ [equation (4)] and *R*_wp_ [equation (5)]; while the background-corrected weighted profile factor *R*_wpb_ [equation (6)] is a better measure of the fitting in the electron diffraction refinements (Luo *et al.*, 2011 ▸). The pure spinel structure (with Al at 8*a* and 16*d* and O at 32*e*) could not produce a good fit, and large errors are found, with *R*_wpb_ = 8.88% (Fig. S2*a* ). After adding the Al 16*c* site to the spinel model (cubic 16*c* model), *R*_wpb_ is reduced to 5.35% (Fig. S2*b* ). The Zhou–Snyder model adds the Al 32*e* to the spinel structure, which gives *R*_wpb_ = 5.79% which is less than that of the spinel but slightly higher than that of the cubic 16*c* structure (Fig. S2*c* ). However, after adding Al 16*c* and 48*f* sites to the spinel structure which becomes the Smrčok model, *R*_wpb_ is significantly reduced to *R*_wpb_ = 3.31%, as shown in Figs. 1 ▸(*c*) and 1 ▸(*d*).

Then, we refine the pattern using the tetragonal Paglia model (Paglia *et al.*, 2003 ▸). A good fit is obtained with *R*_wpb_ = 3.57% [Fig. 1 ▸(*e*)], which is lower than those from the spinel, cubic 16*c* or Zhou–Snyder models. This is consistent with the results using neutron diffraction by Paglia *et al.* (2003 ▸), who observed the same fitting order from spinel model (cubic 1) with the least fit → Zhou–Snyder model → cubic 16*c* model → tetragonal model with the best fit. It is found that the Smrčok model yields a slightly better fit, supporting the results from single-crystal electron diffraction by Ayoola, House *et al.* (2020 ▸). In fact, the Smrčok and Paglia models are closely related, see insets in Figs. S1*a* and S1*b* . The Paglia model is selected with **a** and **b** along two perpendicular ½〈110〉 directions of the cubic structure and **c** remains the same direction. Since *a* and *c* values are nonequal in the tetragonal structure, it offers more flexibilities so better fit could be obtained over the cubic 16*c* or Zhou–Snyder models. However, the Smrčok model contains more Al on the nonspinel tetrahedral 48*f* sites; and during the refinement process, it was found that after adding the 48*f* site, even at very low occupation, the *R* factors could be significantly reduced. Therefore, it gives a clue to modify the tetragonal Paglia model. It is found that after adding the 16*g* (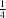, 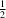, 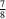) tetrahedral site, surprisingly the *R* factor was significantly reduced, as shown in Fig. 1 ▸(*f*), with *R*_wpb_ = 2.15% which is better that of the Paglia model. As a test, we also added other sites such as tetrahedral 4*b* or 8*e*, and it was found that their refined occupations were almost 0.0, with almost the same *R* factors, so 4*b* and 8*e* are not included in the structure model. The structure of the γ-alumina is refined and the refined parameters are listed in Table 2 ▸.

Considering the Al^3+^ cation fraction at tetrahedral and octahedral positions, as listed in Table 1 ▸, the Smrčok model yields 39% fraction of tetrahedral sites, which is close to the reported value of 37% using the X-ray method (Smrčok *et al.*, 2006 ▸). The refinement using the Paglia model yields 27% tetrahedral sites. By adding 16*g* to the Paglia model, the Al fraction on tetrahedral sites is increased to 35%. From the previous published work, the tetrahedral site fraction was reported as 29% by Paglia *et al.* (2003 ▸), or in the 31–34% range depending on processing temperature in another reference (Paglia *et al.*, 2004 ▸). Rudolph *et al.* (2017 ▸) found 45% Al fraction occupying tetrahedral sites. In fact, the Al^3+^ cation location can be experimentally determined directly using ^27^Al nuclear magnetic resonance (NMR) spectroscopy (Prins, 2020 ▸). Samain *et al.* (2014 ▸) reported the tetrahedral site Al^3+^ fraction as 30%, followed with a report of the fraction in the 35–37% range by Lee *et al.* (2015 ▸), 30–35% range by Khivantsev *et al.* (2020 ▸), 33.7–35.2% range by Huestis *et al.* (2020 ▸), and 39.6% by Xu *et al.* (2021 ▸) recently. The result of 35% fraction of tetrahedral sites by our model is close to those those values reported by the NMR studies.

Considering the Al^3+^ cation fraction on the nonspinel positions (Table 1 ▸), Smrčok *et al.* (2006 ▸) reported that in the γ-alumina single crystals, the Al fraction of nonspinel sites is only 6% according to their supplied online CIF file data; or using the occupation data on the printed page, the fraction is calculated as 14%, because of the very low Al occupation on 16*c* and 48*f* sites. Such a low fraction from single crystals is inconsistent with other results from the boehmite-derived γ-alumina, as questioned by Prins (2020 ▸) recently, since a much higher fraction of nonspinel positions is needed to fit the XRD patterns (Zhou & Snyder, 1991 ▸; Paglia *et al.*, 2003 ▸). However, our refinement yields higher occupations of 16*c* and 48*f* sites than the Smrčok model, giving a higher fraction of 40%. In the literature, the nonspinel position fraction was reported as 25% by Zhou & Snyder (1991 ▸), and 27% by Paglia *et al.* (2003 ▸) experimentally, who further investigated theoretically and predicted over 40% occupation on nonspinel positions (Paglia *et al.*, 2005 ▸). Using atomic pair distribution function analysis of synchrotron powder diffraction data, Samain *et al.* (2014 ▸) found that the nonspinel position fraction is 43–52%. From the refinement results, the nonspinel position fraction is increased from 29% by the Paglia model to 36% by our model (Table 1 ▸), which is consistent with these reports.

### 3.2. Boehmite to γ-alumina *in situ* transformation   

**Figure 2:**
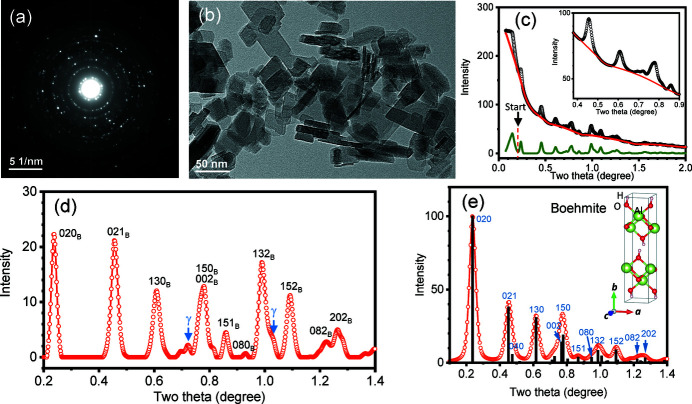
(*a*) Electron diffraction pattern of boehmite; (*b*) image of boehmite crystallites; (*c*) intensity profile of (*a*) and background removal using Brückner algorithm; (*d*) intensity profile after the background removal; (*e*) simulated electron diffraction pattern of boehmite. Arrows in (*d*) indicate the formation of γ-alumina even though the pattern in (*a*) is taken in the beginning of the experiment. Inserted in (*e*) is a structural model of boehmite, where Al is shown in green, O in red, and H in peach (the smallest in size).

During the observation of boehmite nanoparticles, it was noticed that the sample was extremely sensitive to the electron beam, so precautions were made to avoid long exposure to reduce the beam damage. In the experiment, when an area was selected, an electron diffraction pattern was immediately taken [Fig. 2 ▸(*a*)]. The screen current density was 1.0 × 10^−10^ A cm^−2^, and the total time for the sample exposed to the electron beam is estimated as 10 s, so the received dose is estimated as 143.8 × 10^3^ e nm^−2^ (the number of electrons received over 1 nm^2^ area during this period), according to the manufacturer’s specification of the instrument. Afterward, the image was taken [Fig. 2 ▸(*b*)].

Here, we firstly analyze the diffraction pattern in Fig. 2 ▸(*a*). Its intensity profile is shown in Fig. 2 ▸(*c*). We process the background using a simple algorithm proposed by Brückner (2000 ▸), which can provide a quick removal of the high background without the need to refine or simulate the background (Luo, 2016 ▸). The processed background is shown in Fig. 2 ▸(*c*), and an enlargement is shown in the inset. After subtracting the background, the diffraction peaks are obtained, as shown in the enlargement in Fig. 2 ▸(*d*). An electron diffraction pattern of the boehmite structure (Christensen *et al.*, 1982 ▸; Bokhimi *et al.*, 2001 ▸) is simulated in Fig. 2 ▸(*e*) for comparison. It is found that most of the experimental peaks in Fig. 2 ▸(*d*) are consistent with boehmite, while a few small peaks, as indicated by arrows, are from γ-alumina, indicating that although this was the first photo taken in the experiment, the structure was already partially transformed. The lower peak of (020)_B_ in Fig. 2 ▸(*d*), as compared with the simulation, is caused by this background removal method which partially cuts off the peak intensity, as well as the fact that the sample is already partially transformed.

**Figure 3:**
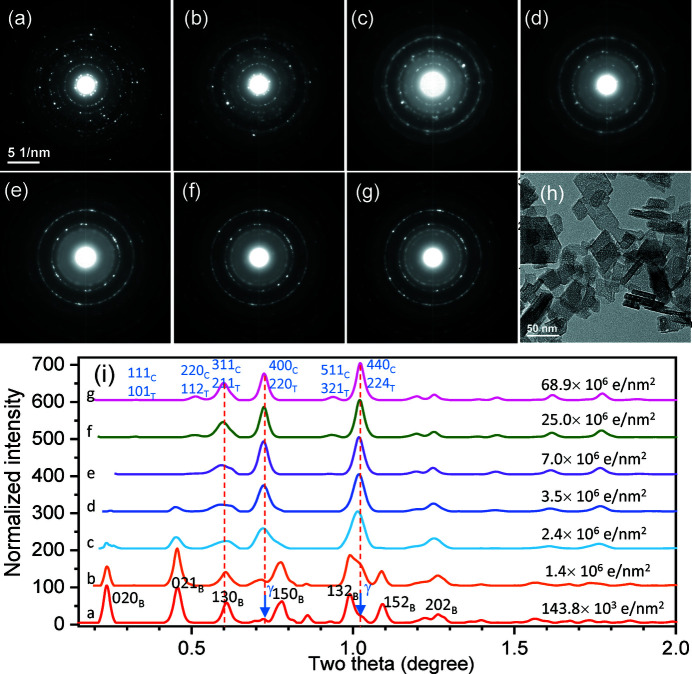
A series of electron diffraction patterns taken from the same area at different dosages. Pattern (*a*) at beginning [same as Fig. 2 ▸(*a*)]; (*b*)–(*d*) after illumination at 100 pA cm^−2^ for an interval of 1 min between them; (*e*) after illumination at 100 pA cm^−2^ for 4 min; (*f*) after illumination at 500 pA cm^−2^ for 4 min; (*g*) after illumination at 500 pA cm^−2^ for 10 min. (*h*) The final image with nanopores after the boehmite to γ transformation and (*i*) intensity profiles of the patterns after background removal, showing the *in situ* transformation process by the e-beam irradiation.

The time taking the image in Fig. 2 ▸(*b*) is estimated as 10 s, and then this specimen area was illuminated for 1 min at the same screen current density of 1.0 × 10^−10^ A cm^−2^, and the second diffraction pattern was taken, as shown in Fig. 3 ▸(*b*) [Fig. 3 ▸(*a*) is the same as Fig. 2 ▸(*a*), which is placed here as a sequence for comparison], where the total dose received is estimated as 1.4 × 10^6^ e nm^−2^. The third [Figs. 3 ▸(*c*)] and fourth [Fig. 3 ▸(*d*)] patterns were also taken in a one minute interval of illumination under the same screen current density of 1.0 × 10^−10^ A cm^−2^, while the fifth [Fig. 3 ▸(*e*)] pattern was taken after four minutes of illumination at the same screen current density of 1.0 × 10^−10^ A cm^−2^. The sixth [Fig. 3 ▸(*f*)] pattern was taken after illuminating at a higher screen current density of 5.0 × 10^−10^ A cm^−2^ for 4 min, and the last one seventh was taken after illuminating for 10 min [Fig. 3 ▸(*g*)] at this high screen current density. The final total dose was estimated as 68.9 × 10^6^ e nm^−2^. As shown in the image in Fig. 3 ▸(*h*), this area is totally damaged, and small pores with 1−2 nm size appear in the crystallites.

After processing in the same way as demonstrated in Fig. 2 ▸, the profiles of these series of diffraction patterns in Figs. 3 ▸(*a*)–3 ▸(*g*) are plotted in Fig. 3 ▸(*i*) for comparison, which clearly shows the boehmite → γ-alumina transformation process. Although the first peak (020)_B_ is interfered by the center beam, the second peak (021)_B_ is evident until the dose of 3.5 × 10^6^ e nm^−2^ (curve d), while the peaks from the γ phase are well developed after 7.0 × 10^6^ e nm^−2^ (curve e). It demonstrates that the Brückner background removal, although it cannot be used for intensity refinement as it generates inaccurate intensities, can provide a quick processing of the high background which always appears in the electron diffraction patterns. However, it should be noted that the irradiated γ-alumina pattern can be indexed as either cubic or tetragonal phases, as indicated on curve g in Fig. 3 ▸(*i*). Therefore, we conduct Rietveld refinement using the last diffraction pattern in Fig. 3 ▸(*g*).

**Figure 4:**
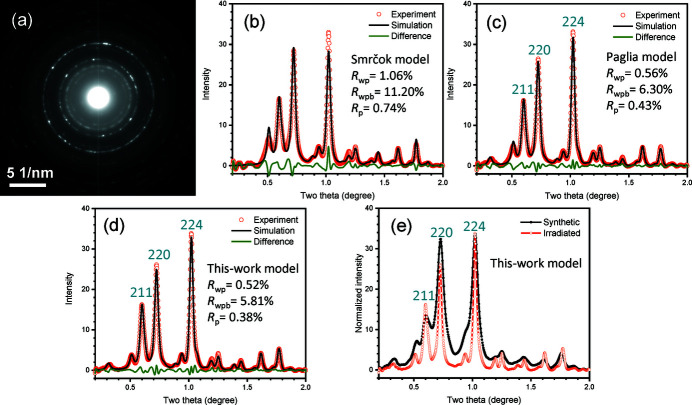
(*a*) The final electron diffraction pattern of γ phase after e-beam irradiation [same as Fig. 3 ▸(*g*)]. (*b*) Rietveld refinement using Smrčok model; (*c*) Rietveld refinement using Paglia model; (*d*) Rietveld refinement using this-work model and (*e*) comparison of synthetic and irradiated γ phase after background removal using the model in this work.

Fig. 4 ▸(*a*) shows the electron diffraction pattern of γ-alumina, derived from boehmite by *in situ* e-beam irradiation [same as Fig. 3 ▸(*g*)]. Rietveld refinements are conducted using different models, as described previously. The Smrčok model could not produce a good fit by maintaining the correct stoichiometry, and a high *R*_wpb_ = 11.20% is obtained [Fig. 4 ▸(*b*)]. The Paglia model could yield a good fit, with *R*_wpb_ = 6.30% [Fig. 4 ▸(*c*)]. However, the model proposed by this work again makes a better fit than the Smrčok and Paglia models, with *R*_wpb_ = 5.81% [Fig. 4 ▸(*d*)]. The refinement results are listed in Table 3 ▸. Compared with Table 1 ▸, it is found that both of the tetrahedral and nonspinel fractions of the irradiated phase are lower than those of the synthetic phase, either by the Smrčok, Paglia or this-work models.

After subtracting the refined background, a comparison of the intensity profiles of the synthetic and irradiated γ-alumina, refined by the model proposed by this work, is shown in Fig. 4 ▸(*e*). It is noted that the irradiated γ-alumina sample exhibits higher (224) over (220) of the tetragonal phase [Fig. 4 ▸(*e*)]. This is due to the fact that the original boehmite crystallites are oriented on the carbon support film [Fig. 2 ▸(*b*)] with a preferred orientation, so the transformed γ-alumina particles are also preferably orientated. Adding the texture in the refinement could reduce the *R* factor, and the refinements in Fig. 4 ▸ indeed include the preferred orientation refinement using March–Dollase approach (Zolotoyabko, 2009 ▸). Despite some intensity differences, the diffraction peaks of the synthetic and irradiated γ-alumina match well, indicating that they have the same type of structure.

### 3.3. Crystallographic orientation and dimensional changes   

**Figure 5:**
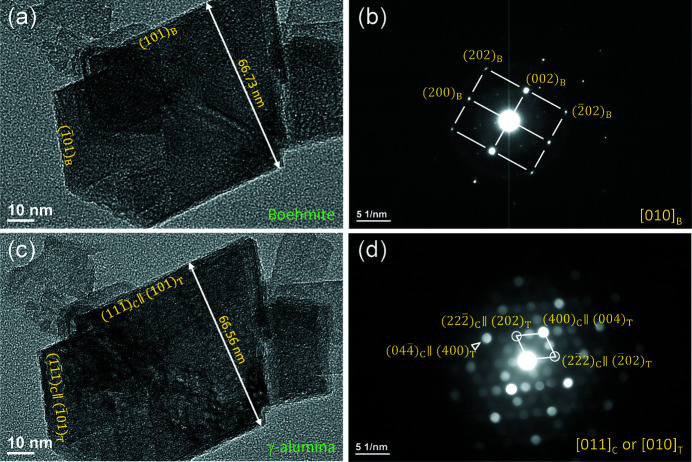
(*a*) Image of boehmite; (*b*) electron diffraction pattern from the boehmite in (*a*); (*c*) image of the crystallite after transformation to γ; (*d*) NBD pattern from the crystallite after transformation.

In order to analyze the crystallographic OR of boehmite and γ-alumina, single boehmite crystallites were selected to observe their *in situ* transformation behavior by e-beam irradiation. Fig. 5 ▸(*a*) shows a boehmite crystallite lying on the carbon support film. The electron diffraction pattern in Fig. 5 ▸(*b*) reveals that it is at the [010]_B_ zone-axis orientation, and its edge surfaces are along (101)_B_ and 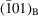. After the beam illumination, the crystallite is quickly damaged with the appearance of nanopores, though they may not extend through the crystallite [Fig. 5 ▸(*c*)], and a nanobeam diffraction (NBD) pattern is taken as shown in Fig. 5 ▸(*d*), which very resembles to cubic [011]_C_, but it also could be indexed as [010]_T_ of the tetragonal structure, as discussed by Paglia *et al.* (2003 ▸). The NBD from a smaller area could provide a better pattern than SAED when the sample is damaged. Simulation of the single-crystal diffraction patterns indicates that the patterns by Smrčok model, Paglia model and model by this work are almost identical in geometry that cannot be visually differentiated without quantitative measurement (Fig. S3). From Figs. 5 ▸(*b*) and 5 ▸(*d*), the following OR is obtained:
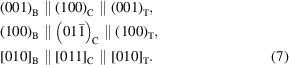
This OR is consistent with the pseudomorphosis relationship between boehmite and cubic structures reported in the literature (Lippens & de Boer, 1964 ▸; Wilson, 1979 ▸; Rudolph *et al.*, 2019 ▸). The width of the crystallite is measured as 66.73 nm before transformation [Fig. 5 ▸(*a*)], and 66.56 nm after the transformation [Fig. 5 ▸(*c*)], and the crystallite shrinks slightly for 0.3%. However, the presence of nanopores interferes with the accurate measurement of the dimensional changes, as the nanopores can accommodate these changes.

**Figure 6:**
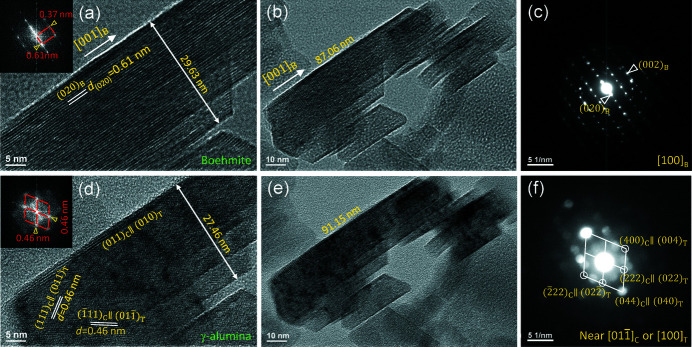
(*a*) Image of boehmite with layers at edge-on orientation, with an insert of its FT pattern; (*b*) image at a lower magnification taken after (*a*); (*c*) electron diffraction pattern from the boehmite crystallite; (*d*) image of the crystallite after transformation to γ, with an insert of its FT pattern; (*e*) image at a lower magnification after the transformation; (*f*) NBD pattern from γ. The crystal pallet shrinks in thickness but expands in length.

Fig. 6 ▸(*a*) is an image of the boehmite crystallite with layers oriented at the edge-on position. Its (020)_B_ layer lattices are clearly visible, with a spacing of 0.61 nm. A Fourier transformation (FT) pattern is inserted. Afterward, an image at a lower magnification is then taken, as shown in Fig. 6 ▸(*b*), where it can be seen from the disappearance of some lattice fringes that the particle is partially damaged. The electron diffraction pattern in Fig. 6 ▸(*c*) confirms the boehmite structure along its [100]_B_ zone axis, with its geometry consistent with the FT pattern in Fig. 6 ▸(*a*). After the beam illumination, the boehmite layers almost completely disappear, replaced with finer lattice fringes with spacing of 0.46 nm, as indicated in Fig. 6 ▸(*d*), while no evident pores are formed yet. If one measures the width, the crystallite shrinks evidently from 29.63 nm to 27.46 nm, with a shrinkage of 7.3%. However, the platelet along its length [001]_B_ direction expands from 87.06 nm [Fig. 6 ▸(*b*)] to 91.15 nm [Fig. 6 ▸(*e*)], with a length change of 4.7%. As the crystal in Fig. 6 ▸(*b*) is already partially transformed, the real expansion from a pure boehmite may be higher than this measurement. After the transformation, the crystallite orientation slightly changes, while the zone axis can be still recognized as 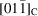 or [100]_T_ from the inserted FT pattern in Fig. 6 ▸(*d*) and NBD pattern in Fig. 6 ▸(*f*). The observed OR is thus
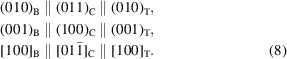



It is noted that although the shrinkage is a simple observation in TEM, to the best of our knowledge, it was not reported in the literature. By *ex situ* measurements, it is hard to find the size changes (Deng *et al.*, 2010 ▸; Peng *et al.*, 2012 ▸; Huestis *et al.*, 2020 ▸). Wilson (1979 ▸) pointed out that no shrinkage was observed along the [010]_B_ orientation, while the layer edge-on orientation was not examined. The dimensional changes will provide evidence regarding the transformation mechanism, which will be discussed in the next section.

**Figure 7:**
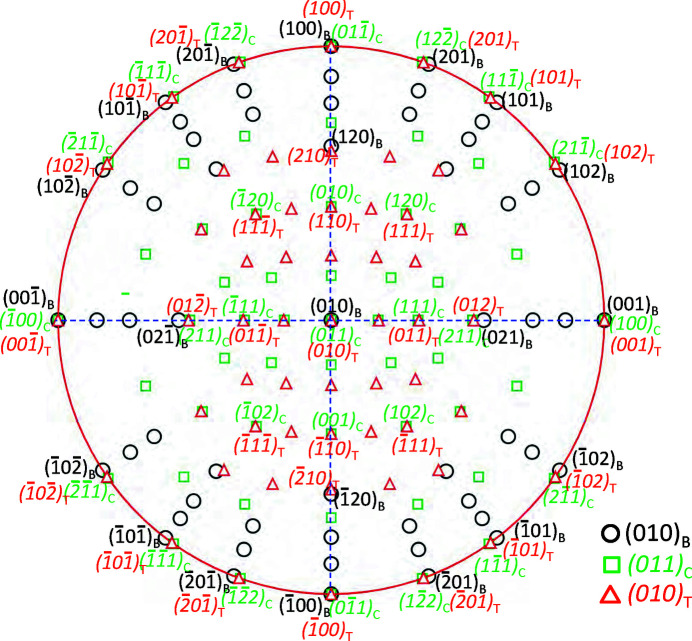
Stereo projection of boehmite, cubic γ and tetragonal γ phases showing their crystallographic orientations.

Based on the above results, the ORs between boehmite, cubic and tetragonal γ-alumina phases are plotted in the stereo-projection in Fig. 7 ▸. It is noted that the boehmite and the tetragonal phase have an axis-on-axis parallel OR. One may use the stereo-projection to analyze their crystallographic orientation and indexing diffraction patterns.

### 3.4. Boehmite to γ-alumina transformation mechanism   

**Figure 8:**
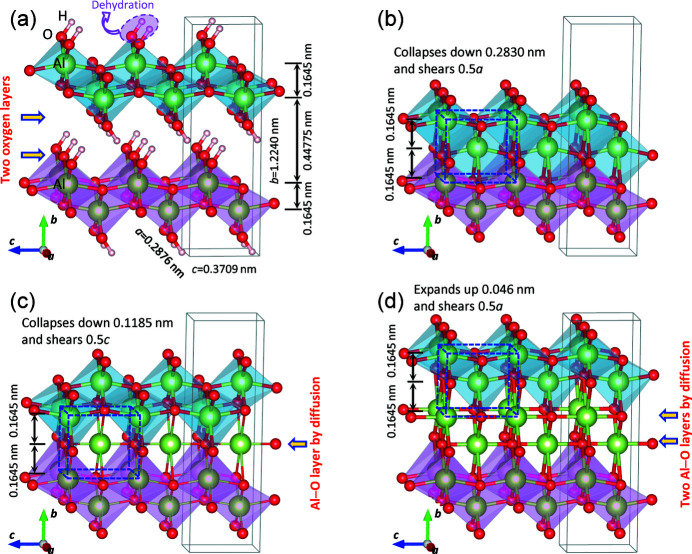
Boehmite to γ-alumina transformation mechanism. (*a*) Boehmite structure (arrows indicate two oxygen layers in the hydroxyl group gap); (*b*) collapse-1 mechanism, where hydroxyl groups are thoroughly removed, and the upper block collapses down with a shear of 0.5*a*; (*c*) collapse-2 mechanism, where after the dehydration, the upper block collapses down with a shearing of 0.5*c*. Only one oxygen layers remains in the gap, which forms an Al–O layer by Al^3+^ diffusion, as indicated by an arrow; (*d*) reaction mechanism, where after the dehydration, the space with two oxygen layers remains filled by diffusion, forming two Al−O layers (indicated by arrows) without the occurrence of collapse. In order to get an intermediate structure for analysis, the upper block slightly expands up with a shearing of 0.5*a*. Outlined in (*c*)–(*d*) are the unit cells of the formed intermediate structure. The upper and lower blocks are shown in different color for clarity, and other polyhedra are not shown.

As mentioned in the introduction, there are two types of boehmite to γ-alumina transformation mechanisms. The collapse mechanism is the commonly accepted one with theoretical investigations (Krokidis *et al.*, 2001 ▸; Nguefack *et al.*, 2003 ▸), and it has been assumed as a default mechanism in the alumina community, though it lacks experimental support. On the other hand, the reaction mechanism receives much less attention and there was no follow-up research, experimentally or theoretically, after Wilson (1979 ▸). Here, we discuss the possible mechanisms from crystallographic point of view, with new insights based on our experimental observations. The transformation mechanisms are shown in Fig. 8 ▸, and the analysis by an atomic projection method (Kuo & Jia, 1985 ▸) is shown in Fig. S4.

As shown in Fig. 8 ▸(*a*), there are two oxygen layers in the hy­droxy group gap, as indicated by arrows. The upper and lower AlO_6_ octahedron blocks are shown in different color for clarity, and the neighboring Al^3+^ layers are spaced with 0.1645 nm. During the dehydration process, as H_2_O molecules by thermal heating (Lippens & de Boer, 1964 ▸; Krokidis *et al.*, 2001 ▸) or H_2_ gas by high-energy irradiation (Westbrook *et al.*, 2015 ▸; Kaddissy *et al.*, 2017 ▸, 2019 ▸; LaVerne & Huestis, 2019 ▸) are removed away from the hydroxyl group gap, there are following possible ways to fill the vacancies as stated below.

**Figure 9:**
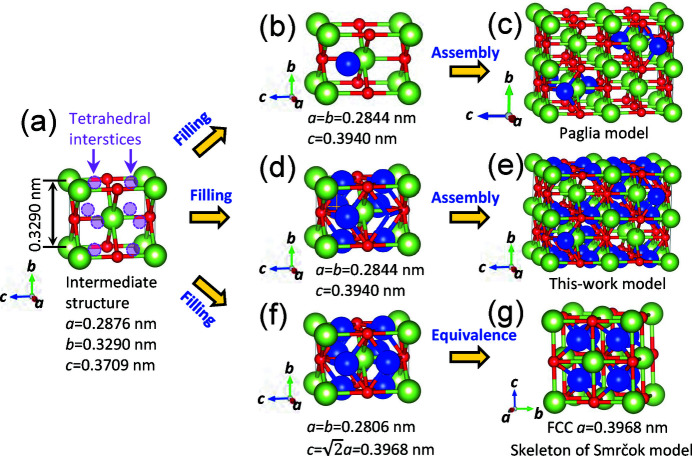
(*a*) Structural model of the intermediate structure, with large available tetrahedral interstices as indicated; (*b*, *c*) filling tetrahedral interstices with a few Al^3+^ cations to form the Paglia model; (*d*, *e*) filling tetrahedral interstices with more Al^3+^ cations to form this-work model; (*f*, *g*) filling tetrahedral interstices with Al^3+^ cations symmetrically to maintain a geometrical constraint (*a* = *b =* 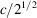) to form a cubic structure, which is a skeleton of the Smrčok model. Notice the substantial lattice contraction along *b* in this process. Octahedral Al is shown in green, tetrahedral Al in blue, and O in red.

(1) Suppose the hydroxyl groups are completely removed from the AlOOH boehmite, and thus the upper block collapses down; while to avoid close contact of Al^3+^ cations, a shear of 0.5*a* (0.1438 nm) occurs [Fig. 8 ▸(*b*)]. This is the most intuitive picture about the collapse which also appears in the literature (for example, in Wilson 1979 ▸). However, it should be noted that the Al and O ratio is terminated as 1:1 rather than 2:3, and the charge is not balanced. Under regular transformation circumstances, it is impossible to occur. It is shown here just for comparison, termed as collapse-1. To keep the Al^3+^ layers with the same spacing of 0.1645 nm, the collapse distance of the upper block is 0.2830 nm, and thus an intermediate structure forms, as outlined in Fig. 8 ▸(*b*).

(2) Suppose H^+^ cations and O^2−^ anions, in a 2:1 ratio, are thoroughly removed and thus only one oxygen layer is remaining in the gap, and Al^3+^ cations nearby diffuse to this oxygen plane to partially fill the large holes, forming an Al–O layer, as indicated by an arrow in Fig. 8 ▸(*c*). The upper block collapses down, with a shearing of 0.5*c* (0.1855 nm) to avoid close contact of Al^3+^ cations, as shown in Fig. 8 ▸(*c*). This is the collapse mechanism generally referred in the literature, which is termed as collapse-2 here. As pointed out by Lippens & de Boer (1964 ▸), one unit cell of boehmite contains four O and four OH layers; after dehydration and collapse, six O layers remain, which form six O layers in the cubic phase that is in a distance of 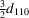 = 0.8421 nm, as indicated in Fig. S4*g* . This collapse is based on the remaining O layers, and only one Al–O layer is formed in the gap after dehydration. According to DFT calculations, Krokidis *et al.* (2001 ▸) also pointed out that Al^3+^ cations move to the gap during structural collapse to form this Al–O layer. With the formation of this Al–O layer, along with collapse and shearing, an intermediate structure forms, as outlined in Fig. 8 ▸(*c*). In fact, this collapse mechanism also involves atomic diffusion.

(3) Alternatively, if the removal of H^+^ and O^2−^ occurs gradually, Al^3+^ and O^2−^ ions nearby immediately diffuse to occupy these vacancies, and thus the hy­droxy group gap remains filled through partially occupation, with no chance to collapse. Instead of one O layer, both two O layers in the gap form Al–O layers through partial occupation by diffusion, as indicated by arrows in Fig. 8 ▸(*d*). This is the reaction mechanism. For our analysis purpose, in order to achieve an intermediate structure with the same layer spacing of 0.1645 nm, the upper structural block expands up slightly along *b* direction for a distance of 0.046 nm, followed with a shearing of 0.5*a* (0.1438 nm) to avoid close contact of Al^3+^ cations. Afterward, an intermediate structure forms, as outlined in Fig. 8 ▸(*d*). Note that although the formation of the intermediate structure in the reaction mechanism requires expansion of the lattice, in the following process, as described in Fig. 9 ▸, substantial contraction occurs from the intermediate structure and the overall length change is still negative along *b* direction. In the real transformation, the expansion and contraction may occur simultaneously, resulting a direct overall contraction.

It is noticed that the intermediate structure formed by the above mechanisms are the same, if only Al^3+^ positions are considered, as shown in Fig. 8 ▸(*b*)−8 ▸(*d*). The partial occupation of Al^3+^ in Figs. 8 ▸(*c*) and 8 ▸(*d*) results in the partial occupation of Al^3+^ in the final γ phase, while the mechanism in Fig. 8 ▸(*b*) does not produce the partial occupation. Further, in the reaction mechanism in Fig. 8 ▸(*d*), after the dehydration the oxygen layers still remain the same, with eight layers per unit cell, suggesting the partial occupation of oxygen in these layers in the initial stage, while eventually as the transformation completes, the oxygen turns to full occupation in the γ phase through the formation of voids, as described by Wilson (1979 ▸).

In the case of the tetragonal γ structure, the pseudomorphosis relationships of the collapse and reaction mechanisms are expressed as
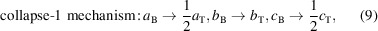


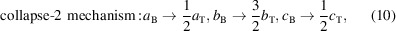


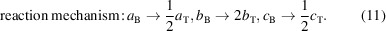



In the case of cubic γ structure, these pseudomorphosis relationships are expressed as
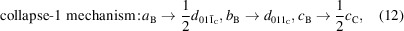


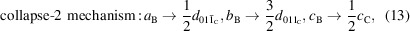


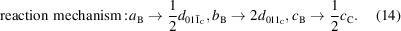



The pseudomorphosis relationships (13) and (14) of the cubic structure are well reported in the literature by Lippens & de Boer (1964 ▸) and Wilson (1979 ▸), respectively. According to these geometric relationships, the dimensional changes of tetragonal and cubic models are listed in Table 4 ▸, based on the assumption that no pores are formed in the crystal. The dimensional changes in *a* and *c* directions are not related to the mechanism. However, the mechanism leads to large differences in the dimension along *b* and also volume. In the tetragonal structure, the collapse-1 and collapse-2 mechanisms produce dimensional changes along *b* as −53.5% and −30.3%, respectively; while the reaction mechanism, only −7.1%. The experimental observation of −7.3% width change in Fig. 6 ▸ supports the reaction mechanism. In addition, the volume change is predicted as high as −51.2% and −26.8% by the collapse-1 and collapse-2, respectively; while by the reaction mechanism, it is only −2.4% without a large change that is consistence with the observation in Fig. 6 ▸. From Table 4 ▸, it is noted that the tetragonal model yields smaller changes in dimensions and volume, compared with the cubic phase. Therefore, the tetragonal structure is more energetically favorable than the cubic phase.

It is noted that our reaction mechanism proposed here involves lattice expansion followed with contraction, which a process is somehow different from the reaction mechanism proposed by Wilson (1979 ▸), who pointed out the idealized reaction mechanism requires pore volume to be ¼ of the original boehmite crystal, the pores occupy ¼ of surface areas. During the transformation induced by e-beam irradiation, the dehydration process may involve the formation of H_2_ produced by the high-energy e-beam irradiation, and probably also with the formation of H_2_O molecules by the e-beam heating effect in the high vacuum; while during the dehydration by conventional calcination to synthesize γ-alumina, only H_2_O molecules are removed. The final products have the same structure, as demonstrated in Fig. 4 ▸. However, the e-beam irradiation enables a direct observation of the transformation in the initial stage. As shown in Fig. 6 ▸, it is observed that the transformation occurs continuously by gradual disappearance of boehmite lattice fringes without the formation of pores in the initial stage, implying that the space filling can occur through partial occupation at this stage, rather than full occupation to form pores. In fact, in the refined structures, the Al^3+^ cations are highly partially occupied. Whereas with high-dose electron illumination, when the transformation completes, partially occupied O anions turn to fully occupied in the lattice by forming nanopores as shown in Fig. 3 ▸(*h*), which is consistent with the observation by Wilson (1979 ▸).

### 3.5. Selection of γ structural models   

The intermediate structure formed in Fig. 8 ▸ is presented in Fig. 9 ▸(*a*), after slight realignment of O positions to symmetrical positions, while the Al^3+^ positions are inherited directly from boehmite. It is a body-centered orthorhombic structure, with dimensions of *a* = 0.2876 nm, *b* = 0.3290 nm, and *c* = 0.3709 nm. In fact, it is a largely distorted NaCl-type structure, as Wilson (1979 ▸) discussed previously. Along [101], 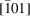 and [001] directions, Al and O are alternatively spaced which is a characteristic of the NaCl-type structure. We use this intermediate structure to further discuss the γ structure models.

This intermediate structure contains large tetrahedral interstice space in this unit cell, as indicated in Fig. 9 ▸(*a*). On the (010) and (100) planes, the distances of the interstice center to Al^3+^/O^2−^ are 0.1711 nm and 0.1888 nm, respectively. Since the radii of Al^3+^ and O^2−^ are 0.053 nm and 0.14 nm, respectively, it is possible to accommodate Al^3+^ in these tetrahedral interstices. In the following process, filling these tetrahedral interstices with lattice distortion constructs different γ-phase structures as follows:

(1) Filling the tetrahedral interstices with a few tetrahedral Al^3+^ cations, with a lattice distortion as indicated in Fig. 9 ▸(*b*), Paglia structure model unit is obtained. Assembly of eight of such units, with different tetrahedral Al^3+^ cations, forms the Paglia model [Fig. 9 ▸(*c*)].

(2) Filling the tetrahedral interstices with more tetrahedral Al^3+^ cations yields a unit for this-work model [Fig. 9 ▸(*d*)]. Again, assembly of eight of such units, with different Al^3+^ cation configurations, produces the model in Fig. 9 ▸(*e*).

(3) Filling the tetrahedral interstices with the tetrahedral Al^3+^ cations in a symmetrically way to maintain a geometric constraint of 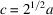 (without the tetragonal distortion), as shown in Fig. 9 ▸(*f*), a face-centered cubic (f.c.c.) structure is obtained, since the unit cell in Fig. 9 ▸(*f*) is equivalent to the f.c.c. structure in Fig. 9 ▸(*g*). It is the skeleton of the Smrčok model, with eight of such units with different assembly of the Al^3+^ cations.

From Fig. 9 ▸, filling the tetrahedral sites with Al cations yields expansion along *c* but substantial contractions along *b*, as listed in Table 5 ▸. The contraction along *b* is so large, causing the overall dimensional change to be negative for the reaction mechanism, even though the lattice slightly expands in the first step [Fig. 8 ▸(*d*)]. During the boehmite → γ-alumina transition, the largest lattice mismatch between the intermediate and γ-alumina phases is along the *b* direction. Comparing the tetragonal structure with the cubic structure, the former one possesses shorter distance along *c* but longer distance along *a* or *b*, *i.e.* its tetragonal distortion (when considered as a near cubic structure after unit-cell conversion) *c*/*a* < 1; therefore, the distortion along *b* direction is reduced. In fact, the lattice distortions along the other *a* and *c* directions are also less than those of the cubic phase (Table 5 ▸).

A recent work indicated that the cations on the tetrahedral positions are moveable (Ayoola, Li* et al.*, 2020 ▸) and they are in a larger fraction on the surfaces. From the refinement in Fig. 4 ▸, it is found that the fractions of tetrahedral and nonspinel positions in the electron-beam irradiated γ-alumina are lower than those in the synthetic alumina. Such a difference is due to the insufficient diffusion during the *in situ* transformation experiment, possibly with insufficient dehydration as well, compared with a standard synthetic route by calcinating boehmite at high temperature of 500–800 °C. Through this transformation mechanism, the resultant γ-alumina crystal structure and ORs with the boehmite are well consistent with the experimental observations.

## 4. Summary   

In summary, we applied quantitative electron microscopy to study the crystal structure of γ-alumina and its transformation mechanisms, and fresh results are obtained as follows.

(1) Based on quantitative Rietveld refinement of electron diffraction patterns using various γ-alumina structural models, better fits were found for cubic Smrčok or tetragonal Paglia models, compared with other cubic or monoclinic models. Among the Smrčok or tetragonal Paglia models, a direct comparison indicated that the former one could provide slightly better or similar fits, although much higher Al fraction was found on nonspinel sites than the report by Smrčok *et al.* (2006 ▸). A new structure model was proposed for the γ-alumina by adding a 16*g* site to the tetragonal structure, which could provide better fitting over the current models.

(2) By electron beam irradiation, boehmite to γ-alumina *in situ* transformation was observed as a function of electron dose. The transformed γ phase was identified to be of the same type of structure as the synthetic phase, although with less fractions of tetrahedral and nonspinel cations, due to insufficient diffusion in the *in situ* transformation process.

(3) In the literature, the two boehmite → γ-alumina transformation mechanisms were not clearly described. The commonly accepted collapse mechanism is based on the assumption that H^+^ and O^2−^ (in 2:1) are thoroughly removed to free the space for collapsing [collapse-2 in Fig. 8 ▸(*c*)], while the reaction mechanism is based on the assumption that the dehydrated gap maintains filled through Al and O diffusion without collapsing [Fig. 8 ▸(*d*)]. Using the concept of an intermediate structure, we provided new insights into these two types of mechanisms and discussed their transformation process in detail, with expected dimensional and volume changes provided after the transformation. Our proposed reaction mechanism involves lattice expansion along *b* to form an intermediate structure, followed with substantial contraction to form the γ structure. From the *in situ* observation of single boehmite crystallites, evident shrinkage of 7.3% along boehmite *b* direction was directly observed. This observation supports the reaction mechanism with a good agreement, rather than the commonly regarded collapse mechanism which requires a much higher shrinkage and a large volume contraction. Further, the observation of gradual disappearance of boehmite lattice fringes suggested the occurrence of reaction mechanism through partial occupation of the dehydrated gap in the initial stage of the transformation, without the formation of voids as proposed by Wilson (1979 ▸). The voids only appeared as the transformation continued after the initial stage.

(4) With the aid of intermediate structure, the formation of different γ-alumina structures was discussed by filling the tetrahedral interstices with Al^3+^ cations of the intermediate structure. Among them, the tetragonal structure requires smaller lattice distortion than the cubic structure, meaning that it is more energetically favorable than the cubic structure. The proposed mechanisms yield ORs with the parent boehmite phase that are consistent with the experimental observations.

## Supplementary Material

Crystal structure: contains datablock(s) I. DOI: 10.1107/S2052520621008027/je5042sup1.cif

Fig S1-S4. DOI: 10.1107/S2052520621008027/je5042sup2.pdf

CCDC reference: 2101470

## Figures and Tables

**Table 1:** Rietveld refinement results of electron diffraction pattern from synthetic γ-alumina nanoparticles using different structural models

Model	Unit-cell parameter (nm)	Space group	Atomic site: occupation	Tetrahedral fraction (reference data) (%)	Nonspinel fraction (reference data) (%)	*R*_wp_ (%)	*R*_wpb_ (%)	*R*_p_ (%)
Pure spinel	*a* = 0.7959 (4)	Fd\bar 3m	O 32*e*: 1.000 Al 8*a*: 0.771 (5), 16*d*: 1.000	28 [28 in Paglia *et al.* (2003 ▸)]	0 [0 in Paglia *et al.* (2003 ▸)]	1.37	8.88	0.98
Cubic 16*c*	*a* = 0.7960 (2)	Fd\bar 3m	O 32*e*: 1.000 Al 8*a*: 0.727 (5), 16*d*: 0.592 (8), 16*c*: 0.372 (4)	27 [29 in Paglia *et al.* (2003 ▸)]	28 [45 in Paglia *et al.* (2003 ▸)]	0.55	5.35	0.42
Zhou–Snyder	*a* = 0.7950 (2)	Fd\bar 3m	O 32*e*: 1.000 Al 8*a*: 0.730 (6), 16*d*: 0.586 (9), 32*e*: 0.201 (3)	27 [31 in Zhou & Snyder (1991 ▸)]	30 [25 in Zhou & Snyder (1991 ▸)]	0.53	5.79	0.40
Smrčok	*a* = 0.7935 (1)	Fd\bar 3m	O 32*e*: 1.000 Al 8*a*: 0.505 (3), 16*d*: 0.548 (4), 16*c*: 0.273 (2), 48*f*: 0.087 (1)	39 [37 in Smrčok *et al.* (2006 ▸)]	40 [6–14 in Smrčok *et al.* (2006 ▸)]	0.37	3.31	0.27
Paglia	*a* = 0.5690 (2), *c* = 0.7880 (5)	*I*4_1_/*amd*	O 16*h*: 1.000 Al 4*a*: 0.710 (4), 8*c*: 0.378 (3), 8*d*: 0.591 (5)	27 [29 in Paglia *et al.* (2003 ▸)]	29 [27 in Paglia *et al.* (2003 ▸); >40 in Paglia *et al.* (2005 ▸)]	0.38	3.57	0.31
This work	*a* = 0.5687 (1), *c* = 0.7880 (2)	*I*4_1_/*amd*	O 16*h*: 1.000 Al 4*a*: 0.486 (3), 8*c*: 0.263 (1), 8*d*: 0.611 (3), 16*g*: 0.109 (1)	35	36	0.20	2.15	0.15

**Table 2:** Refined structure of γ-alumina Space group: *I*4_1_/*amd* (#141, origin choice 2); unit-cell parameters: *a* = 0.5687 (1) nm, *c* = 0.7880 (2) nm; *R* factors: *R*_wp_ = 0.20%, *R*_wpb_ = 2.15%, *R*_p_ = 0.15%

Atom	Wyckoff site	*x*	*y*	*z*	*B*_iso_ (Å^2^)	Occupation
O	16*h*	0	0.0113 (4)	0.2646 (4)	1.0	1.000
Al	4*a*	0	0.75	0.125	0.3	0.486 (3)
Al	8*c*	0	0	0	0.3	0.263 (1)
Al	8*d*	0	0	0.5	0.3	0.611 (3)
Al	16*g*	0.25	0.5	0.875	0.3	0.109 (1)

**Table 3:** Rietveld refinement results of electron diffraction pattern from γ-alumina nanoparticles derived from boehmite by electron beam irradiation

Model	Unit-cell parameter (nm)	Space group	Atomic site: occupation	Tetrahedral fraction (reference data) (%)	Nonspinel fraction (reference data) (%)	*R*_wp_ (%)	*R*_wpb_ (%)	*R*_p_ (%)
Smrčok	*a* = 0.7942 (2)	Fd\bar 3m	O 32*e*: 1.000 Al 8*a*: 0.725 (7), 16*d*: 0.67 (1), 16*c*: 0.247 (4), 48*f*: 0.020	32 [37 in Smrčok *et al.* (2006 ▸)]	23 [6–14 in Smrčok *et al.* (2006 ▸)]	1.06	11.20	0.74
Paglia	*a* = 0.5620 (1), *c* = 0.7971 (3)	*I*4_1_/*amd*	O 16*h*: 1.000 Al 4*a*: 0.545 (3), 8*c*: 0.257 (3), 8*d*: 0.814 (5)	20 [29 in Paglia *et al.*, (2003 ▸)]	19 [27 in Paglia *et al.* (2003 ▸); >40 in Paglia *et al.* (2005 ▸)]	0.56	6.30	0.43
This work	*a* = 0.5615 (1), *c* = 0.7988 (2)	*I*4_1_/*amd*	O 16*h*: 1.000 Al 4*a*: 0.457 (4), 8*c*: 0.229 (3), 8*d*: 0.712 (5), 16*g*: 0.082 (4)	29	30	0.52	5.81	0.38

**Table 4:** Changes in dimensions through the boehmite → γ-alumina transformation based on assumption no pores are present in the crystal (length unit: nm)

Model	Direction	Unit-cell parameter γ	Matching distance in γ	Boehmite	Change indimension (%)	Change of volume (%)
Tetragonal	*a* direction	0.5687	0.2844	0.2876	−1.1	−51.2 (collapse-1 mechanism); −26.8 (collapse-2 mechanism); −2.4 (reaction mechanism)
	*b* direction in collapse-1 mechanism	0.5687	0.5687	1.2240	−53.5	
	*b* direction in collapse-2 mechanism	0.5687	0.8531	1.2240	−30.3	
	*b* direction in reaction mechanism	0.5687	1.1374	1.2240	−7.1	
	*c* direction	0.7880	0.3940	0.3709	6.2	
Cubic	*a* direction	0.7935	0.2805	0.2876	−2.5	−52.2 (collapse-1 mechanism); −28.3 (collapse-2 mechanism); −4.3 (reaction mechanism)
	*b* direction in collapse-1 mechanism	0.7935	0.5611	1.2240	−54.2	
	*b* direction in collapse-2 mechanism	0.7935	0.8416	1.2240	−31.2	
	*b* direction in reaction mechanism	0.7935	1.1222	1.2240	−8.3	
	*c* direction	0.7935	0.3968	0.3709	7.0	

**Table 5:** Lattice mismatch of intermediate structure with γ phase models

Model	Direction	Intermediate structure	γ phase	Mismatch (%)
Tetragonal	*a* direction	0.2876	0.2844	−1.1
	*b* direction	0.3290	0.2844	−13.6
	*c* direction	0.3709	0.3940	6.2
Cubic	*a* direction	0.2876	0.2805	−2.5
	*b* direction	0.3290	0.2805	−14.7
	*c* direction	0.3709	0.3968	7.0
