# Supplementary material for: Structure of boehmite-derived γ-alumina and its transformation mechanism revealed by electron crystallography
Source: Acta Crystallogr B Struct Sci Cryst Eng Mater. 2021 Sep 16;77(Pt 5):772–84. doi: 10.1107/S2052520621008027 (PMC8489438; doi:10.1107/S2052520621008027)
Supplement: Supplementary file 2 [file b-77-00772-sup2.pdf]

STRUCTURAL SCIENCE
CRYSTAL ENGINEERING
MATERIALS

Volume 77 (2021)

Supporting information for article:

Structure of boehmite-derived γ-alumina and its transformation mechanism revealed by electron crystallography

Zhiping Luo

Supporting Information

Structure of boehmite-derived γ-alumina and its transformation mechanism revealed by electron crystallography

Zhiping Luo

Department of Chemistry, Physics and Materials Science, Fayetteville State University, Fayetteville, NC 28301, USA

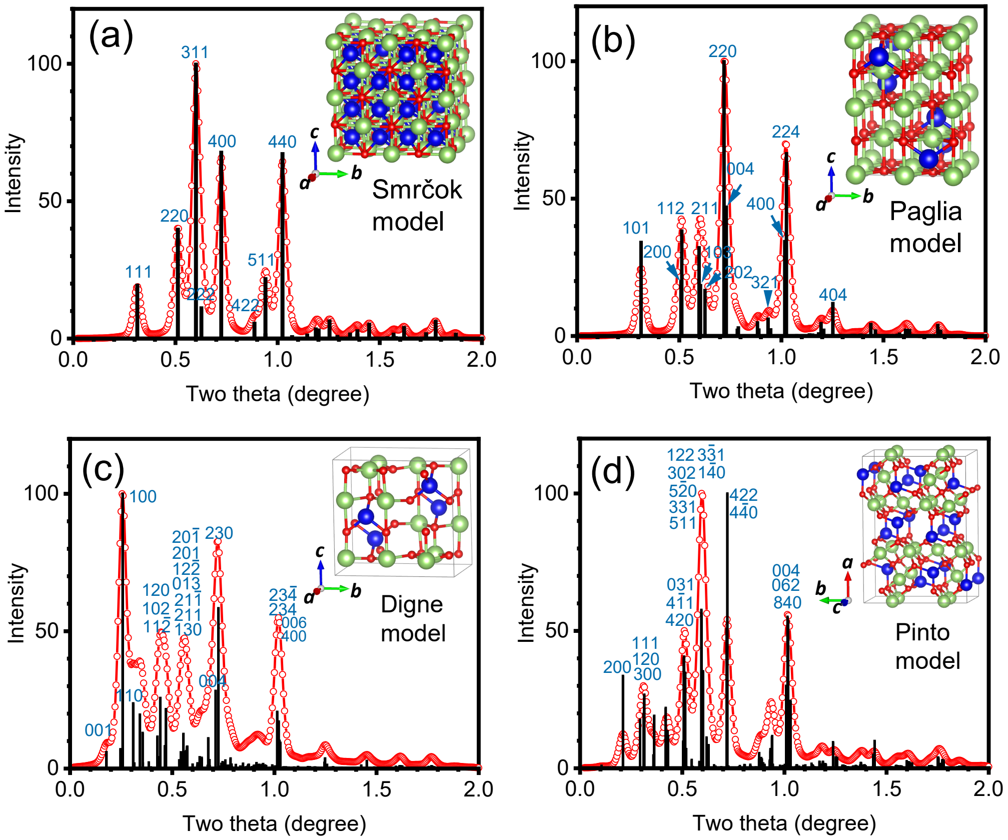

Fig. S1. Simulated electron diffraction patterns using cubic Smrčok model (a), tetragonal Paglia model (b), monoclinic Digne model (c), and monoclinic Pinto model (d). Structural models are inserted (green: octahedral Al; blue: tetrahedral Al; red: oxygen).

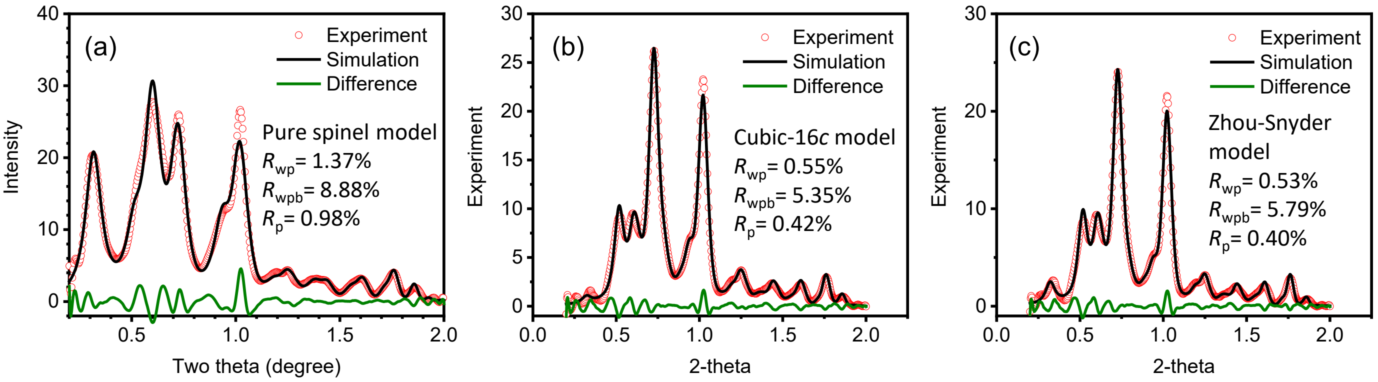

Fig. S2. Rietveld refinement of electron diffraction patterns from synthesized γ-alumina using different structural models: (a) pure cubic spinel model (Al at 8*a* and 16*d* sites); (b) Cubic-16c model by adding Al at 16*c* site to the spinel model (Al at 8*a*, 16*d* and 16*c* sites); and (c) Zhou-Snyder model (Al at 8*a*, 16*d* and 32*e* sites).

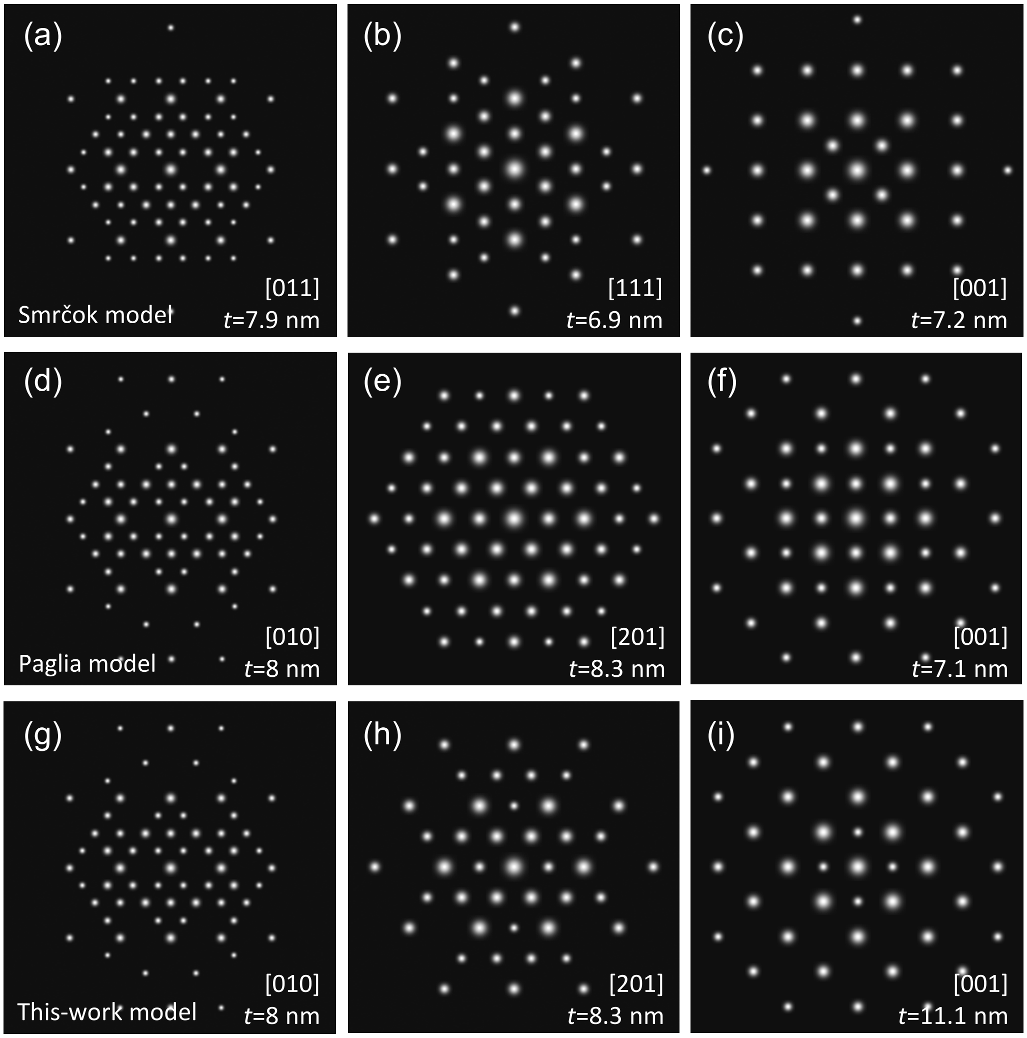

Fig. S3. Simulated single-crystal electron diffraction patterns along three major zone axes using Smrčok model (a–c), Paglia model (d–f) and this-work model (g–i). The thickness is indicated for each pattern.

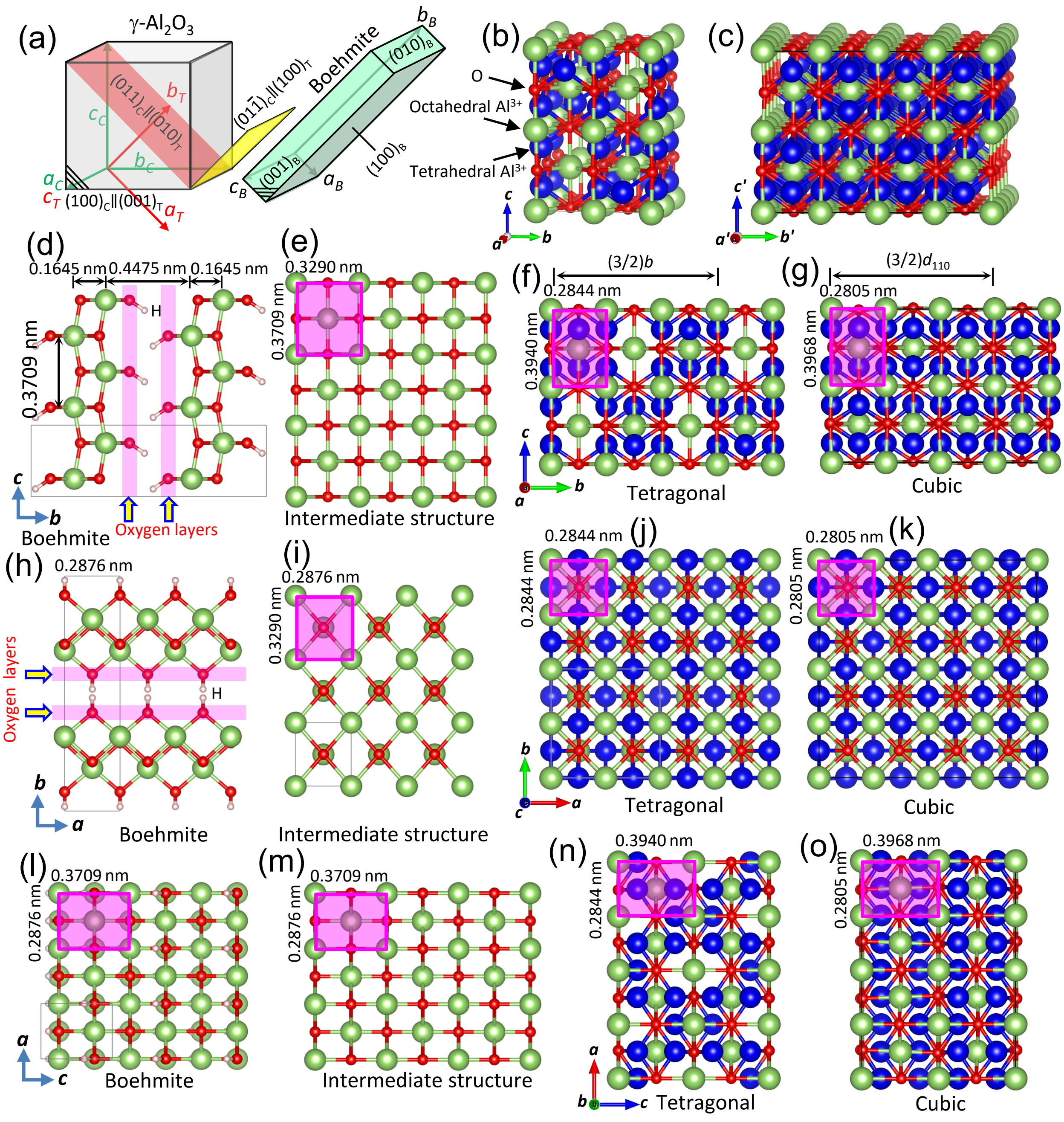

Fig. S4. Atomic projections. (a) Orientations between boehmite, cubic γ and tetragonal γ phases; (b) structural model of the tetragonal model proposed by this work; (c) structural model of cubic Smrčok model after conversion; (d–g) atomic projections along [100] direction of boehmite, intermediate structure, tetragonal γ and cubic γ; (h–k) atomic projections along [001] direction of boehmite, intermediate structure, tetragonal γ and cubic γ; (l–o) atomic projections along [010] direction of boehmite, intermediate structure, tetragonal γ and cubic γ. For the γ phase structure, octahedral Al is shown in green, and tetrahedral Al in blue.
